# An Assessment of Knowledge and Awareness of Human Trafficking Among Health Care Professionals and Students in Texas, 2020

**DOI:** 10.1177/00333549251361335

**Published:** 2025-09-12

**Authors:** Esther Nwokocha, Ya-Ching Huang, Bruce Machona, Janice Hernandez, Adam Blank, Li-Chen Lin

**Affiliations:** 1 School of Nursing, The University of Texas at Austin, Austin, TX, USA; 2 College of Nursing, Texas A&M University, College Station, TX, USA

**Keywords:** human trafficking, health care professionals, awareness, recognition, nursing education, survivor care

## Abstract

**Objectives:**

Human trafficking results in negative health outcomes among survivors. However, a lack of awareness and understanding of human trafficking limits health care professionals’ ability to provide appropriate care in primary and community care settings. We assessed the general understanding and awareness of human trafficking among students, staff, and faculty at The University of Texas at Austin.

**Methods:**

We used self-report instruments in an online survey to collect data on demographic characteristics and responses to questions on human trafficking knowledge, awareness, and the needs of survivors. We summarized participants’ knowledge, awareness of, training on, and exposure to institutional protocols related to human trafficking and examined subgroup differences. We also analyzed participants’ perspectives on awareness, training, and protocols in responses to open-ended items.

**Results:**

Most respondents were female (80 of 85; 94.1%) and associated with the School of Nursing (84 of 85; 98.8%). The average for correct responses to knowledge of human trafficking was 70%. Few respondents (n = 11; 13%) had received at least 1 hour of training in human trafficking in the last year. Most respondents (n = 57; 67.1%) were unsure about ways to request training on human trafficking and whether their institution had any structure for addressing it. Not knowing how to assist human trafficking victims without putting them in danger was reported as difficult. A lack of knowledge and training was reported as a barrier to identifying victims.

**Conclusions:**

Limited understanding and knowledge related to human trafficking and a lack of protocols for nursing professionals to address human trafficking are barriers to effective health care provision. Regular education for students and health care professionals would provide much-needed support for human trafficking survivors.

Human trafficking, the recruitment, transportation, transfer, harboring, or receipt of a person by threat or force to have control over that person for the purpose of exploitation, is a global threat to human rights and health.^1^ In the United States, an estimated 313 000 people are trafficked in Texas alone, 79 000 of whom are minors (aged <18 y) engaged in sex trafficking and 234 000 of whom are subjected to labor trafficking.^2^ Although efforts have been made to prevent human trafficking in the United States and other countries, its effects on survivors are a persistent concern.^1,3,4^ Despite increased support during the past decade for training health care professionals to address human trafficking, gaps remain in efforts to educate the public and health care workers about this offense and how to prevent it.^5,6^

Human trafficking is evolving into a market-driven industry that exploits an estimated 40.3 million people globally.^7,8^ Reasons for human trafficking include involuntary prostitution, forced marriage, organ transplants, abduction of children, and forced labor.^5,7,9,10^ To meet the demand for cheap labor, human trafficking victims are essentially modern-day slaves. Traffickers experience little to no deterrence in their trade, owing to governments’ and law enforcement’s lack of capacity to identify and investigate it effectively.^11^ In addition, trauma-informed community awareness is insufficient, and resources for prevention and services for recovery are limited.^12^ Children are the most vulnerable population targeted for human trafficking^13^; about 1.2 million children worldwide are coerced and lured into this rapidly growing industry annually.^1^ Children who experience homelessness and/or have mental health diagnoses, substance misuse, or involvement with child protective services are especially at risk of human trafficking.^1,14^ Despite the prevalence of human trafficking, health care professionals often lack the tools needed to identify and assist human trafficking survivors effectively because of gaps in knowledge about their roles in addressing this issue.^15^ This lack of awareness and understanding of risk factors for human trafficking limits health care providers’ ability to identify and provide appropriate care to survivors in multiple settings,^4^ highlighting the need for further education. Training that incorporates an evidence-based, person-centered, trauma-informed approach is therefore essential for educating current and future health care professionals on human trafficking.^6^

Health care settings frequently serve as critical points of contact for human trafficking survivors, because these individuals often seek treatment for physical or psychological consequences of trafficking, such as trauma-related injuries, migraines, pain, stomach ulcers, chronic illnesses, or mental disorders.^5,16^ Despite the necessity for comprehensive services and a continuum of care,^17^ many human trafficking victims’ needs are unmet. Emergency departments, clinics, and community health care centers are common entry points for trafficked individuals, but health care providers face barriers to recognizing and supporting them. Barriers include limited training, unclear reporting protocols, and fear of endangering the survivor.^18^ Enhancing nurses’ education and awareness of human trafficking is crucial to address these gaps and improve the support and treatment of human trafficking victims.

Although extensive research has emphasized the importance of human trafficking education for clinicians, less is known about the knowledge and awareness levels of future health care professionals and the faculty responsible for their training.^18^ We investigated the current understanding of human trafficking among students, staff, and faculty at The University of Texas at Austin (UT). By focusing on academic environments, we aimed to identify educational gaps that could inform targeted curriculum development and improve future health care responses to human trafficking. The consequences of human trafficking in the lives of survivors demand that health care professionals address and proactively understand it. The objective of this study was to assess human trafficking awareness and knowledge about responding to human trafficking to inform the training of current and future health care professionals. We explored the health care profession’s understanding of its role in preventing, identifying, rescuing, and treating human trafficking survivors.

## Methods

### Study Design

We used a descriptive, cross-sectional design and an online survey to assess knowledge, awareness, and training needs related to human trafficking among participating university students, faculty, and staff in an academic setting. We administered the survey via Qualtrics from January to September 2020. Participant recruitment started after approval from UT’s institutional review board, which determined this study was exempt from review. We recruited eligible participants from the university’s professional schools or colleges of nursing, pharmacy, social work, communications, and medicine. To be eligible for inclusion, participants had to be aged ≥18 years, able to read and write in English, and currently enrolled or employed at the university. Recruitment efforts included mass email distributions, flyers, and direct outreach to faculty teaching courses related to health care ethics and human trafficking to encourage students’ participation. Each school or college’s offices for administration and student services distributed the recruitment flyer and a recruitment message via email with a survey link. The survey included questions on demographic characteristics, questions extracted from a study by Ross et al^4^ that estimated what proportion of National Health Service professionals in the United Kingdom had encountered trafficked people and measured their knowledge and confidence to respond to human trafficking, and questions developed after a review of the literature.

### Measures

#### Demographic information

Demographic data comprised participants’ self-reported age, sex, race and ethnicity, school or department, and position in the school or department.

#### Actual knowledge about human trafficking

Nineteen survey items assessed factual knowledge of human trafficking; the items were adapted for US health care practices from the scale developed by Ross et al^4^ for health care professionals in the United Kingdom’s National Health Service. The survey question about the prevalence of criminal human trafficking was modified to reflect US information. Participants answered yes, no, or not sure to each question. Responses were scored as correct or incorrect. Scores ranged from 0 to 19; higher scores indicated higher human trafficking knowledge levels. Internal consistency for this scale in the present study was good, with a Cronbach α of .80.

#### Perceived readiness, awareness, training, and protocols

This section of the survey included 21 questions that evaluated self-reported familiarity with human trafficking concepts and perceived readiness to respond to survivors. To assess the response to human trafficking in health care, the research team developed 5 items based on a review of the literature in addition to 13 questions by Busch-Armendariz et al.^2^ It included questions on prior human trafficking training, the availability of institutional resources, and perceived barriers to assisting survivors. Examples included the following: “How knowledgeable do you consider yourself in the area of human trafficking?” Answers ranged from “knowledgeable” to “not at all knowledgeable.” To understand gaps in current education needs, 3 open-ended questions were included at the end of the multiple-choice questions, such as, “In your opinion, what are the biggest barriers to identifying victims of human trafficking?” (eFigure in Supplemental Material).

This study was approved by the Institutional Review Board of the Office of Research Support and Compliance at UT (no. 2020-01-0002). All participants gave written informed consent prior to participating in the study.

### Data Analysis

We used SPSS version 25 (IBM Corp) and descriptive statistics to analyze and describe data. We used frequency distributions and percentages to summarize participants’ actual knowledge about human trafficking and perceived knowledge, awareness, training, and protocols related to human trafficking. We used the 1-way analysis of variance test to explore differences in knowledge across subgroups (faculty, staff, and students). For participant responses to the open-ended questions in the awareness, training, and protocols measurement, 2 authors independently read and analyzed qualitative responses. The first author (E.N.) and last author (L.-C.L.) then conducted thematic data analysis to further explore emerging themes, nuances, and meanings. Sentences and phrases were coded to describe the meaning expressed in the data. Common themes were identified and compared for consensus.

## Results

The survey was sent to approximately 200 potential respondents across the 5 schools; 111 people participated in the survey. After removing responses with >5% missing data, 85 respondents remained for final analysis (42.5% response rate after excluding incomplete surveys). The sample included a mix of undergraduate (n = 31; 36.5%) and graduate (n = 16; 18.8%) students, faculty (n = 19; 22.4%), and staff (n = 19; 22.4%) (Table 1). Most respondents were female (n = 80; 94.1%) and non-Hispanic White (n = 46; 54.1%). Eighty-four (98.5%) respondents reported that they were associated with the School of Nursing or identified nursing as their professional discipline. Although all health professional schools were approached, the analysis, results, and discussion focus on respondents associated with the School of Nursing.

**Table 1.** Characteristics of student, staff, and faculty respondents (N = 85) to a survey on human trafficking knowledge, awareness, and response to the needs of human trafficking survivors, at the University of Texas at Austin, Texas, 2020

Characteristic	No. (%)
Sex	
Female	80 (94.1)
Male	4 (4.7)
Other	1 (1.2)
Age, y	
18-24	35 (41.2)
25-30	7 (8.2)
31-40	11 (12.9)
41-50	19 (22.4)
51-60	6 (7.1)
61-70	5 (5.9)
≥71	2 (2.4)
Race and ethnicity	
African American/Black	3 (3.5)
Asian	13 (15.3)
Hispanic/Latino	19 (22.4)
White	46 (54.1)
Mixed	3 (3.5)
School/department	
Nursing	84 (98.8)
Social work	1 (1.2)
Position in the school/department	
Faculty	19 (22.4)
Staff	19 (22.4)
Undergraduate student	31 (36.5)
Graduate student	16 (18.8)

### Actual Knowledge About Human Trafficking

The average rate of correct responses was 70.7% (range, 15.3%-98.8%) (Table 2). The 3 items with the most frequent incorrect or “do not know” answers were the following: “coronary heart disease is not likely to be related to situations of human trafficking” (44.7% incorrect; 40.0% did not know), “Diabetes is not likely to be related to situations of human trafficking” (37.6% incorrect; 42.4% did not know), and “Calling the police if I suspect a patient has been trafficked could put the patient in more danger” (40.0% incorrect; 31.8% did not know). We found no significant differences in median actual knowledge score about human trafficking among faculty (14.26), staff (12.68), undergraduate students (13.16), and graduate students (13.81) (*P* = .43).

**Table 2.** Actual knowledge about human trafficking among student, faculty, and staff respondents (N = 85) to a survey on human trafficking knowledge, awareness, and response to the needs of human trafficking survivors the University of Texas at Austin, Texas, 2020

True/false survey statements	No. (%) answering correctly	No. (%) answering incorrectly	No. (%) answering “do not know”
1. Definition of human trafficking is restricted to women and girls who have been trafficked into prostitution.	80 (94.1)	1 (1.2)	4 (4.7)
2. In 2018, over half (51.6%) of the criminal human trafficking cases active in the US were sex trafficking cases involving only children.	38 (44.7)	18 (21.2)	29 (34.1)
3. The majority of women who are trafficked for prostitution were sex workers before being trafficked.	74 (87.1)	3 (3.5)	8 (9.4)
4. Children who are working for relatives in domestic situations cannot really be considered “trafficked.”	72 (84.7)	0 (0.0)	13 (15.3)
5. Trafficking is associated with posttraumatic symptoms.	78 (91.8)	2 (2.4)	5 (5.9)
6. Trafficking is associated with chronic headaches.	48 (56.5)	5 (5.9)	32 (37.6)
7. There are usually evident signs that a person is in a trafficking situation.	54 (63.5)	19 (22.4)	12 (14.1)
8. People who are being exploited often have difficulty reporting these situations to outsiders, especially professionals.	84 (98.8)	0	1 (1.2)
9. Health practitioners should not ask trafficked people about violence that they might have suffered, as it is too traumatic for them.	65 (76.5)	5 (5.9)	15 (17.6)
10. Calling the police if I suspect a patient has been trafficked could put the patient in more danger.	24 (28.2)	34 (40.0)	27 (31.8)
11. Depression is not likely to be related to situations of human trafficking.	78 (91.8)	2 (2.4)	5 (5.9)
12. Chemical burns and pesticide poisoning are not likely to be related to situations of human trafficking.	55 (64.7)	2 (2.4)	28 (32.9)
13. Memory problems are not likely to be related to situations of human trafficking.	68 (80.0)	4 (4.7)	13 (15.3)
14. Coronary heart disease is not likely to be related to situations of human trafficking.	13 (15.3)	38 (44.7)	34 (40.0)
15. Diabetes is not likely to be related to situations of human trafficking.	17 (20.0)	32 (37.6)	36 (42.4)
16. Hypothermia and dehydration are not likely to be related to situations of human trafficking.	72 (84.7)	1 (1.2)	12 (14.1)
17. Sexually transmitted infections are not likely to be related to situations of human trafficking.	79 (92.9)	1 (1.2)	5 (5.9)
18. Headaches are not likely to be related to situations of human trafficking.	60 (70.6)	1 (1.2)	24 (28.2)
19. Posttraumatic stress disorder is not likely to be related to situations of human trafficking.	82 (96.5)	1 (1.2)	2 (2.4)

### Perceived Readiness, Awareness, Training, and Protocols in Human Trafficking

Most participants reported that they were either somewhat knowledgeable (n = 34; 40.0%) or slightly knowledgeable (n = 28; 32.9%), and 15.3% were very knowledgeable (n = 2) or knowledgeable (n = 11) about perceived knowledge about human trafficking, Most participants considered staff and coworkers at their agency or facility to be somewhat knowledgeable (n = 36; 42.4%) or slightly knowledgeable (n = 29; 34.1%) about human trafficking; 15.3% were very knowledgeable (n = 1) or knowledgeable (n = 12). Almost all participants perceived sex trafficking (n = 82; 96.5%) and labor trafficking (n = 74; 87.1%) as a bigger problem than most people think. Forty-eight respondents (56.5%) said they were not sure whether their agency or facility considered itself to be informed about trauma from human trafficking. Only 11 participants (12.9%) had received at least 1 hour of training specific to human trafficking in the last year. Fifty-seven (67.1%) respondents were not sure from whom to request training for human trafficking in their agency or whether their agency had any guides, manuals, protocols, or policies related to human trafficking. Fear of not knowing what to say to victims and how to provide them with help without putting them in danger were frustration points or difficulties in providing care to human trafficking victims. Lack of knowledge, lack of training, and ignorance were reported as barriers to identifying human trafficking victims.

Respondents’ open-ended responses reinforced key findings from the survey. Symptoms of posttraumatic stress disorder emerged as common signs and symptoms of human trafficking victims observed by respondents; they included anxious behavior, fear, unusual quietness, unexplainable behaviors, and younger age of patients with multiple sexually transmitted diseases. Two of the most frustrating and difficult aspects of providing care to human trafficking victims were the lack of initiatives from health care professionals to report and rescue victims and victims’ reluctance to leave their situation, probably as a result of Stockholm syndrome whereby victims were never left alone for private conversations. Lack of knowledge, support, training, resources to provide help, and awareness among health care providers, as well as slowness in responses from available resources, stigma, volume of trauma, and difficulty in victims’ identification and assessment, emerged as barriers to identifying victims. About 31% of participants indicated that lack of knowledge or awareness and lack of training were the 2 biggest barriers to identifying human trafficking victims. Other barriers included victims’ vulnerability due to poverty, dependency, wars, and global unrest. One respondent added that human traffickers use businesses to cover up human trafficking activities. One participant, a mother, stated, “We just found out [the foot massage place] is being used for sex trafficking. My teen daughter has been walking unsupervised past that place for months . . . and we had no idea—scary.”

## Discussion

Consistent with other studies,^19^ gaps in knowledge and a lack of confidence in responding to human trafficking prevailed among study participants. An understanding of how much health care professionals know about human trafficking and of their current involvement, awareness, and contact with survivors is crucial. Findings from this study shed light on the gaps in knowledge and awareness of the complex and multifaceted issues related to human trafficking. These findings are applicable to nurses and students, especially those with minimal to no training in human trafficking.

### Actual Knowledge About Human Trafficking

The average knowledge score (70.6%) on our survey suggests a moderate understanding of human trafficking concepts, with substantial misconceptions about the health consequences of trafficking and appropriate responses to suspected cases. For example, only 28.2% of participants correctly identified that contacting law enforcement without survivor consent could increase risk. All participants perceived that sex trafficking (96.5%) and labor trafficking (87.1%) were bigger problems than most people think. Further studies are needed to explore comparable results in other health care facilities and health professional schools.

To address this knowledge gap, health care professionals need additional training on human trafficking. As in the study by Ross et al,^4^ participants in our study reported insufficient training in how to assist individual human trafficking survivors. People who attend training are more likely than those without training to recognize a trafficked person.^4,15^ Powell et al^6^ suggested a process for curricula development and training of health care professionals. Emphasizing departments with high exposure to human traffickers or trafficked individuals, particularly emergency and pediatrics, is vital for enhancing knowledge of and improving recognition and reporting of human trafficking.^20^ Grace et al^21^ underscored that human trafficking victims are most frequently encountered in emergency departments or facilities but also present in other settings such as clinics.^18,22^

### Perceived Readiness, Awareness, Training, and Protocols

Although most respondents recognized the severity of sex and labor trafficking, only 13% reported receiving training on human trafficking in the past year. Most respondents were unsure about institutional policies (56.5%) and were unaware of available resources to address human trafficking (67.1%), underscoring the need for clearer guidelines and access to trauma-informed care frameworks. These findings align with the findings of Testa^19^ that nurses and allied health care professionals may hesitate to report suspected cases because of concerns that reporting without clear policies and procedures could endanger trafficked individuals. In the study by Testa, participants identified training and tools for screening and identification as the top 2 priorities for effectively serving human trafficking victims. Health care professionals expressed frustration and difficulty in providing care due to fear of saying the wrong thing to victims and uncertainty about how to assist without putting them at further risk. In a literature review, Sousou et al^23^ identified challenges faced by healthcare professionals in identifying and reporting potential human trafficking cases. Raker^17^ emphasized the need to incorporate human trafficking–related content into nursing curricula at all levels to better prepare health care professionals to recognize and address human trafficking.

The negative consequences of human trafficking among survivors can manifest physically, psychologically, emotionally, mentally, and/or spiritually.^8,24^ Health care professionals must address both the immediate effects of human trafficking and the long-term health of survivors by providing continuous access to health care.^10,25,26^ Education and advocacy are crucial to achieving this objective.^4,27^ Although studies have underscored the pivotal role of health care professionals in responding to human trafficking, few health professional schools or colleges include human trafficking in their curricula.^2^ Training on human trafficking can enhance trainees’ knowledge and awareness of human trafficking,^28^ and well-structured programs can improve learners’ knowledge, awareness, and attitudes about human trafficking.^29,30^ Health care agencies and educational institutions should consider incorporating such training and education into their curricula and professional development initiatives. Progress has been made; for example, the Texas legislature mandated human trafficking training as part of the continuing competency requirements for nurses renewing their licenses on or after September 1, 2020.^31^ To address these global issues, it is imperative that those in all areas of health care actively participate once they are professionally trained.

### Limitations

This study had several limitations. First, the study had a small sample size. Second, the inclusion of students, faculty, and academic staff, rather than practicing clinicians, restricted generalizability to nonclinical environments. Future studies should include practicing clinicians in health care settings, where encounters with human trafficking survivors are more likely to occur. Third, participants were from a single university, 98.8% of participants were from the School of Nursing, and only 1 participant was from the School of Social Work. Future research should consider more study sites and disciplines to increase the sample’s diversity and size to allow a more comprehensive examination of differences in human trafficking education needs across various demographic groups and disciplines.

### Implications

Our findings provide insights for curriculum development. Unfortunately, human traffickers are getting smarter and trickier to identify, and more sophisticated and updated screening tools should be made available. Health professional schools must prioritize human trafficking education early in training to ensure that future clinicians are equipped. Recommended strategies include the following:

- incorporating case-based learning and simulations into coursework to enhance practical skills;
- establishing interprofessional training modules that address the complex needs of human trafficking survivors; and
- developing clear, accessible institutional policies to guide responses to suspected trafficking cases.

Although this study was based in the United States, its findings align with international research on human trafficking education. In the United Kingdom, for example, human trafficking training has been integrated into competency-based nursing curricula, with an emphasis on trauma-informed care.^4^ Similar approaches in the United States, tailored to local prevalence and health care systems, could strengthen global responses to human trafficking.

## Conclusions

Human trafficking is a critical public health challenge that requires a multifaceted response. By identifying gaps in knowledge and training in academic environments, this study lays the groundwork for targeted educational interventions that can improve care for human trafficking survivors. The students, faculty, and staff of the School of Nursing showed limited familiarity with issues related to human trafficking, and they reported a need for increased training to improve awareness and knowledge about human trafficking to improve care. In addition to education and identification, various barriers may make it even more challenging to help this marginalized trafficked population. The topic of human trafficking is relevant to educators at the college level and to health care organizations. The development of educational resources and curriculum related to human trafficking is needed. Clear policies and protocols to follow, with step-by-step processes from identification through discharge and engagement with community services, could better prepare health care in response to human trafficking. Human trafficking may be closer to us than we think, given the accessibility of technology among young people and the proliferation of hazardous social media applications. Yet properly responding to human trafficking, knowing when to call law enforcement, and knowing how to make referrals to support services may seem overwhelming. It is therefore crucial for health care professionals to first recognize it in addressing this violation of human rights. Expanding this research to clinical settings and diverse populations will further illuminate the path to a comprehensive, trauma-informed health care response.

## Supplemental Material

sj-docx-1-phr-10.1177_00333549251361335 – Supplemental material for An Assessment of Knowledge and Awareness of Human Trafficking Among Health Care Professionals and Students in Texas, 2020 Supplemental material, sj-docx-1-phr-10.1177_00333549251361335 for An Assessment of Knowledge and Awareness of Human Trafficking Among Health Care Professionals and Students in Texas, 2020 by Esther Nwokocha, Ya-Ching Huang, Bruce Machona, Janice Hernandez, Adam Blank and Li-Chen Lin in Public Health Reports®
