# Supplementary material for: An Assessment of Knowledge and Awareness of Human Trafficking Among Health Care Professionals and Students in Texas, 2020
Source: Public Health Rep. 2025 Sep 12;140(5-6):577–83. doi: 10.1177/00333549251361335 (PMC12432013; doi:10.1177/00333549251361335)
Supplement: Supplementary file 1 [file sj-docx-1-phr-10.1177_00333549251361335.docx]

**Perceived readiness, awareness, training, and protocols**

**The following questions relate to knowledge and training in human trafficking.**

1. How knowledgeable do you consider YOURSELF in the area of human trafficking?

- Very Knowledgeable
- Knowledgeable
- Somewhat Knowledgeable
- Not Very Knowledgeable
- Not at All Knowledgeable

2. How knowledgeable do you consider the staff/coworkers at your agency/facility in the area of human trafficking?

- Very Knowledgeable
- Knowledgeable
- Somewhat Knowledgeable
- Not Very Knowledgeable
- Not at All Knowledgeable

3. Does your agency/facility consider itself to be trauma-informed? This includes staff, programs, services, or approach to serving victims.

- Yes
- No
- Not sure

4. Please provide some examples of trauma-informed programs, services, or approach to serving victims.

5. Approximately how many trainings specific to human trafficking did agency staff receive within the last year?

6. Who from your agency attended the training(s)? Select all that apply.

- All staff
- Supervisors
- Frontline staff
- Temporary staff, such as interns and volunteers
- Other, please specify: ____________________
- No training offered

7. Does your agency know where to access that training curriculum or who to request training from if training is needed for human trafficking?

- Yes
- No
- Not sure

8. How would you rate the seriousness of the sex trafficking problem in your area?

- Not a Problem
- Minor Problem
- Moderate Problem
- Serious Problem
- Very Serious Problem

9. Do you think that sex trafficking is a bigger problem than most people think?

- Yes
- No
- Not sure

10. How would you rate the seriousness of the labor trafficking problem in your area?

- Not a Problem
- Minor Problem
- Moderate Problem
- Serious Problem
- Very Serious Problem

11. Do you think that labor trafficking is a bigger problem than most people think?

- Yes
- No
- Not sure

12. When thinking about the number of labor trafficking victims in your service area, select the statement you agree with most.

- We serve all of the victims in our service area
- There are AS MANY victims who we don't see compared to those we serve
- There are TWICE as many victims who we don't see compared to those we serve
- There are THREE TIMES as many victims who we don't see compared to those we serve
- There are FOUR TIMES as many victims who we don't see compared to those we serve
- There are FIVE OR MORE TIMES as many victims who we don't see compared to those we serve
- I do not know
- We are not a provider for human trafficking victims

13. Thinking about your agency, please describe any innovative programs or activities specific to human trafficking that you have not already listed in previous questions.

14. Does your agency have any guides, manuals, protocols, or policies related to human trafficking?

- Yes
- No
- Not sure

15. Would your agency be willing to share any guides, manuals, protocols, or policies related to human trafficking with the research team?

- Yes
- No
- Not sure

16. Think about a time when you suspected that a patient in your care was a victim of human trafficking. What made you suspicious?

17. What was the biggest frustration or most difficult part of providing care to victim of human trafficking?

18. Please rank order the resources needed to better serve victims of human trafficking (with #1 being the top priority).

______ Training

______ Procedures / Protocols

______ Tools (such as for screening or identification)

______ Funding

______ Personnel / Volunteers

______ Other, please specify:

20. In your opinion, what are the biggest barriers to identifying victims of human trafficking?

21. Please share any additional information not covered in previous questions

We appreciate your participation in this survey. Please select NEXT to submit your answers.
